# Supplementary material for: Prognostic factors associated with failure of total elbow replacement: a protocol for a systematic review
Source: BMJ Open. 2023 Aug 30;13(8):e071705. doi: 10.1136/bmjopen-2023-071705 (PMC10471856; doi:10.1136/bmjopen-2023-071705)
Supplement: Supplementary data [file bmjopen-2023-071705supp001.pdf]

Supplementary File 1

Electronic search strategies

MEDLINE search - Ovid interface

#	Query
1	exp Arthroplasty, Replacement, Elbow/
2	total elbow replac*.mp. [mp=title, book title, abstract, original title, name of substance word, subject heading word, floating sub-heading word, keyword heading word, organism supplementary concept word, protocol supplementary concept word, rare disease supplementary concept word, unique identifier, synonyms]
3	total elbow arthropl*.mp. [mp=title, book title, abstract, original title, name of substance word, subject heading word, floating sub-heading word, keyword heading word, organism supplementary concept word, protocol supplementary concept word, rare disease supplementary concept word, unique identifier, synonyms]
4	(total and elbow and replac*).ab.
5	(total and elbow and replac*).ti.
6	(total and elbow and arthropl*).ab.
7	(total and elbow and arthropl*).ti.
8	((elbow adj2 arthroplast*) or (elbow adj2 replac*)).mp.
9	1 or 2 or 3 or 4 or 5 or 6 or 7 or 8

EMBASE search- Ovid interface

#	Query
1	exp elbow replacement/ or exp elbow arthroplasty/
2	total elbow replac*.mp. [mp=title, abstract, heading word, drug trade name, original title, device manufacturer, drug manufacturer, device trade name, keyword heading word, floating subheading word, candidate term word]
3	total elbow arthropl*.mp. [mp=title, abstract, heading word, drug trade name, original title, device manufacturer, drug manufacturer, device trade name, keyword heading word, floating subheading word, candidate term word]

4	(total and elbow and replac*).ab.
5	(total and elbow and replac*).ti.
6	(total and elbow and arthropl*).ab.
7	(total and elbow and arthropl*).ti.
8	((elbow adj2 arthroplast*) or (elbow adj2 replac*)).mp.
9	1 or 2 or 3 or 4 or 5 or 6 or 7 or 8
10	limit 9 to conference abstract status
11	9 not 10

PubMed search

ID	Search
#1	"Arthroplasty, Replacement, Elbow"[Mesh]
#2	total elbow replac*<br><br>("total"[All Fields] OR "totaled"[All Fields] OR "totaling"[All Fields] OR "totalled"[All Fields] OR "totalling"[All Fields] OR "totals"[All Fields]) AND ("elbow"[MeSH Terms] OR "elbow"[All Fields] OR "elbow joint"[MeSH Terms] OR ("elbow"[All Fields] AND "joint"[All Fields]) OR "elbow joint"[All Fields] OR "elbow s"[All Fields] OR "elbows"[All Fields]) AND "arthropl*"[All Fields]
#3	total elbow arthropl*<br><br>("total"[All Fields] OR "totaled"[All Fields] OR "totaling"[All Fields] OR "totalled"[All Fields] OR "totalling"[All Fields] OR "totals"[All Fields]) AND ("elbow"[MeSH Terms] OR "elbow"[All Fields] OR "elbow joint"[MeSH Terms] OR ("elbow"[All Fields] AND "joint"[All Fields]) OR "elbow joint"[All Fields] OR "elbow s"[All Fields] OR "elbows"[All Fields]) AND "replac*"[All Fields]
#4	"total"[Title/Abstract] AND "elbow"[Title/Abstract] AND "replac*"[Title/Abstract]
#5	"total"[Title/Abstract] AND "elbow"[Title/Abstract] AND "arthropl*"[Title/Abstract]
#6	(elbow n2 arthropl*) OR (elbow n2 replac*)<br><br>(("elbow"[MeSH Terms] OR "elbow"[All Fields] OR "elbow joint"[MeSH Terms] OR ("elbow"[All Fields] AND "joint"[All Fields]) OR "elbow joint"[All Fields] OR "elbow s"[All Fields] OR "elbows"[All Fields]) AND "n2"[All Fields] AND "arthropl*"[All Fields]) OR (("elbow"[MeSH Terms] OR "elbow"[All Fields] OR "elbow joint"[MeSH Terms] OR ("elbow"[All Fields] AND "joint"[All Fields]) OR "elbow joint"[All Fields] OR "elbow s"[All Fields] OR "elbows"[All Fields]) AND "n2"[All Fields] AND "replac*"[All Fields])
#9	1 or 2 or 3 or 4 or 5 or 6

Cochrane library search

ID	Search
#1	MeSH descriptor: [Arthroplasty, Replacement, Elbow] explode all trees
#2	total elbow replac*
#3	total elbow arthropl*
#4	(Total) AND (Elbow) AND ("replac*"or"arthropl*") (Word variations have been searched)
#5	#1 OR #2 OR #3 OR #4
