# Supplementary material for: Prognostic factors associated with failure of total elbow replacement: a protocol for a systematic review
Source: BMJ Open. 2023 Aug 30;13(8):e071705. doi: 10.1136/bmjopen-2023-071705 (PMC10471856; doi:10.1136/bmjopen-2023-071705)
Supplement: Author's manuscript [file bmjopen-2023-071705.draft_revisions.pdf]

BMJ Open is committed to open peer review. As part of this commitment we make the peer review history of every article we publish publicly available.

When an article is published we post the peer reviewers' comments and the authors' responses online. We also post the versions of the paper that were used during peer review. These are the versions that the peer review comments apply to.

The versions of the paper that follow are the versions that were submitted during the peer review process. They are not the versions of record or the final published versions. They should not be cited or distributed as the published version of this manuscript.

BMJ Open is an open access journal and the full, final, typeset and author-corrected version of record of the manuscript is available on our site with no access controls, subscription charges or pay-per-view fees (http://bmjopen.bmj.com).

If you have any questions on BMJ Open's open peer review process please email info.bmjopen@bmj.com

BMJ Open

Prognostic factors associated with failure of total elbow replacement: a protocol for a systematic review

Journal:	*BMJ Open*
Manuscript ID	bmjopen-2023-071705
Article Type:	Protocol
Date Submitted by the Author:	17-Jan-2023
Complete List of Authors:	Hamoodi, Zaid; The University of Manchester Centre for Epidemiology Versus Arthritis, Centre for Musculoskeletal Research; Wrightington Wigan and Leigh NHS Foundation Trust, Upper Limb Unit<br>Gehringer, Celina; The University of Manchester Centre for Epidemiology Versus Arthritis, Centre for Musculoskeletal Research, University of Manchester<br>Bull, Lucy; Health Navigator Ltd<br>Hughes, Tom; Manchester Metropolitan University Department of Health Professions; Manchester United Ltd<br>Kearsley-Fleet, Lianne ; The University of Manchester Centre for Epidemiology Versus Arthritis, Centre for Musculoskeletal Research, The University of Manchester<br>Watts, Adam; Wrightington Wigan and Leigh NHS Foundation Trust, Upper Limb Unit<br>Sergeant, Jamie; The University of Manchester Centre for Epidemiology Versus Arthritis, Centre for Musculoskeletal Research, Manchester Academic Health Science Centre,; The University of Manchester, Centre for Biostatistics, School of Health Sciences, Faculty of Biology, Medicine and Health, Manchester Academic Health Science Centre
Keywords:	ORTHOPAEDIC & TRAUMA SURGERY, Adult orthopaedics < ORTHOPAEDIC & TRAUMA SURGERY, Elbow & shoulder < ORTHOPAEDIC & TRAUMA SURGERY, Musculoskeletal disorders < ORTHOPAEDIC & TRAUMA SURGERY

SCHOLARONE™
Manuscripts

Title page

Title of the article:

Prognostic factors associated with failure of total elbow replacement: a protocol for a systematic review

Full name, postal address and e-mail of the corresponding author:

Zaid Hamoodi

Office 2.612, Second floor, Stopford building, Centre for Epidemiology Versus Arthritis, Division of Musculoskeletal and Dermatological Sciences, University of Manchester, UK

Zaid.hamoodi-2@postgrad.manchester.ac.uk

Full name, department, institution, city and country of all co-authors

- Mr Zaid Hamoodi. 1) Centre for Epidemiology Versus Arthritis, Centre for Musculoskeletal Research, University of Manchester, Manchester Academic Health Science Centre, Manchester, UK 2) Upper Limb Unit, Wrightington Hospital, Wrightington, Wigan and Leigh Teaching Hospitals NHS Foundation Trust, Wigan, UK
- Miss Celina K Gehringer. Centre for Epidemiology Versus Arthritis, Centre for Musculoskeletal Research, University of Manchester, Manchester Academic Health Science Centre, Manchester, UK Miss Lucy M Bull. Health Navigator Ltd, London, UK
- Miss Lucy M Bull. Health Navigator Ltd, London, Greater London, UK
- Dr Tom Hughes. 1) Department of Health Professions, Manchester Metropolitan University, Manchester, UK 2) Manchester United Ltd, Manchester, UK
- Dr Lianne Kearsley-Fleet. Centre for Epidemiology Versus Arthritis, Centre for Musculoskeletal Research, University of Manchester, Manchester Academic Health Science Centre, Manchester, UK
- Dr Jamie C Sergeant. 1) Centre for Biostatistics, School of Health Sciences, Faculty of Biology, Medicine and Health, University of Manchester, Manchester Academic Health Science Centre, Manchester, UK 2) Centre for Epidemiology Versus Arthritis, Centre for Musculoskeletal Research, University of Manchester, Manchester Academic Health Science Centre, Manchester, UK

For peer review only

- Professor Adam C Watts. Upper Limb Unit, Wrightington Hospital, Wrightington, Wigan and Leigh Teaching Hospitals NHS Foundation Trust, Wigan, UK

Word count, excluding title page, abstract, references, figures and tables

2679 words

Keywords

Arthroplasty, Replacement, Elbow, Prognosis, Prognostic factors

For peer review only

Abstract

Introduction

Total elbow replacement (TER) has higher failure rates requiring revision surgery compared with the replacement of other joints. Understanding the factors associated with failure is essential for informed decision-making between patients and clinicians, and for reducing the failure rate. This review aims to identify, describe, and appraise the literature examining prognostic factors for failure of TER.

Methods and analysis

This systematic review will be conducted and reported in line with the PRISMA-P guidelines. Electronic literature searches will be conducted using Medline, EMBASE, PubMed, and Cochrane. The search strategy will be broad, including a combination of subject headings (MESH) and free text search. This search will be supplemented with a screening of reference lists of the included studies and relevant reviews. Two independent reviewers will screen all search results in two stages (title and abstract, and full text) based on the PICOTS criteria. The types of evidence included will be randomised trials, non-randomised trials, prospective and retrospective cohort studies, registry studies, and case-control studies. If the literature lacks enough studies, then case series with 50 or more TERs will be considered for inclusion. Data extraction and risk of bias assessment for included studies will be performed by two independent reviewers using the CHARMS-PF and QUIPS tools.

Meta-analyses of prognostic estimates for each factor will be undertaken for studies that are deemed to be sufficiently robust and comparable. Several challenges are likely to arise due to heterogeneity between studies, therefore, subgroup and sensitivity analyses will be performed to account for the differences between studies. Heterogeneity will be assessed using Q and I^2 statistics. If I^2 >40% then pooled estimates will not be reported. When quantitative synthesis is not possible, a narrative synthesis will be undertaken. The quality of the evidence for each prognostic factor will be assessed using the GRADE tool.

For peer review only

Strengths and limitations of this study

- This is the first systematic review to focus on identifying and appraising studies evaluating prognostic factors associated with failure of total elbow replacement.
- This review will follow the methodological advances in performing systematic reviews and meta-analyses in prognosis research proposed by the PROGnosis REsearch Strategy (PROGRESS) and The Cochrane Prognosis Methods Group (PMG).
- This review will use the recommended checklists and tools including the Patient, Intervention, Comparison, Outcome, Time, and Setting (PICOTS) model for study selection, the Checklist for Critical Appraisal and Data Extraction for Systematic Reviews of Prediction Modelling Studies for Prognostic Factors (CHARMS-PF) for data extraction, and the Quality In Prognostic Studies (QUIPS) tool to assess the risk of bias.
- This review may be limited by the quality of and the heterogeneity between the included studies. We aim to address this by performing statistical analysis of heterogeneity combined with subgroup and sensitivity analyses.

For peer review only

For peer review only - http://bmjopen.bmj.com/site/about/guidelines.xhtml

INTRODUCTION

Total elbow replacement (TER) is a recognised treatment for painful arthritic elbow and whilst rheumatoid arthritis remains the main indication, it is increasingly performed for acute trauma and post-traumatic arthritis[1]. Despite technological advances, TER has higher failure rates than other total joint replacements, with a reported 5-year failure rate of 6-10% and 10-year failure rate of 15-19% compared to a 3% 5-year failure rate and <5% 10-year failure rate in total hip replacement [1-8]. In the event of TER failure, surgical revision is usually required but this procedure carries a risk of adverse events such as ongoing pain, disruption of the elbow extensor mechanism, reduction of bone stock, infection, ulnar nerve injury, and poor function[9]. Understanding the factors associated with failure is essential to inform the development of strategies designed to reduce failure risk and the need for subsequent revision surgery, which is a burden for both patients and society[10].

A prognostic factor is defined as any variable that is associated with a risk of a health outcome among people with a particular health condition, in this context the outcome is failure, and the condition is patients with TER[11 12]. Several existing studies have examined possible factors that may be associated with the risk of failure in TER, including primary pathology, prosthesis type, surgeon's experience, implant fixation, and whether surgery is performed in a specialised centre[2 13-19]. However, some of the findings have been inconsistent. For example, some studies examining the association between the underlying pathology and TER failure found rheumatoid arthritis to be associated with a worse prognosis while others found post-traumatic arthritis to be associated with a worse prognosis[6 16].

To the best of our knowledge, there are no systematic reviews available that have appraised, pooled, and synthesised all existing studies relating to prognostic factors for TER failure, which means that the quality of the current evidence base is unknown. This also means that it is unclear whether potential factors have been derived from single exploratory studies (thus having provisional prognostic value) or replicated and confirmed in multiple studies (thus demonstrating robust prognostic value).

For peer review only - http://bmjopen.bmj.com/site/about/guidelines.xhtml

Therefore, the purpose of this systematic review is to identify, describe, appraise, and synthesise all the current literature examining the prognostic factors associated with failure of TER. A better understanding of prognostic factors may help to explain the differences in risk of failure between patients[20], which may facilitate decisions about whether or not to proceed with TER as a definitive management option. An improved understanding of prognostic factors may also help reduce the number of potential TER revisions by improving treatment selection[21] and may help to identify areas for novel interventions[11]. This review will also identify gaps in the evidence base to aid the planning of future research and could potentially pave the way to developing a prognostic model in TER that could be used to make individualised patient predictions.

METHODS AND ANALYSIS

A systematic review will be conducted and reported in line with the Preferred Reporting Items for Systematic reviews and Meta-Analyses (PRISMA-P) guidelines[22]. It has been registered at the International prospective register of systematic reviews (PROSPERO) and will follow the published guidance and recommendation produced by the PROGnosis REsearch Strategy (PROGRESS) and The Cochrane Prognosis Methods Group (PMG) on how to undertake a systematic review and meta-analysis in prognostic factors research[11 12 20 23-25].

Patients and Public Involvement

The study aim was co-designed with the Wrightington Hospital Patients and Public Involvement (PPI) group, including members with long-term health conditions and experience of joint replacement surgery. The group also contributed to a list of possible prognostic factors to be included in the review and their ongoing involvement will help ensure that the review is relevant to patients.

Study eligibility

All peer-reviewed studies will be included if prognostic factors are investigated in patients who have undergone a primary TER, and if the selection criteria in Table 1 are met (based on the Patient, Intervention, Comparison, Outcome, Time, and Setting (PICOTS) model)[12]. Studies must include a human population and provide a quantitative result or give tabulated

For peer review only - http://bmjopen.bmj.com/site/about/guidelines.xhtml

individual patient data (IPD) evaluating prognostic factors for failure of total elbow replacement. This initial list of candidate prognostic factors to be considered are listed in Table 2 but, importantly, this is not exhaustive and studies will also be included if other candidate prognostic factors have been investigated. Although the primary interest is in prognostic factors measured before surgery, we will not exclude prognostic factors measured intraoperatively or postoperatively.

The outcome of failure will be defined as revision surgery. The definition of revision surgery varies between joint registries and is likely to vary between other non-registry studies[26], therefore, a broad definition for revision surgery will be used which is any secondary surgery to the prosthesis, this includes addition, removal, or alterations to all or part of the construct on the same elbow occurring at any time following the primary total elbow replacement. This may lead to the inclusion of studies that evaluate non-revision secondary surgeries as part of a composite outcome. If this occurs, these studies will undergo subgroup/sensitivity analyses.

The types of evidence included will be randomised trials, non-randomised trials, prospective and retrospective cohort studies, registry studies, and case-control studies. If the literature is lacking in studies of these types, then a case series with 50 TERs or more will be considered for inclusion. Results from case series will be interpreted with caution due to the higher risk of bias, especially selection bias. Review articles, surveys, case reports, and conference abstracts will be excluded.

If the literature is published in a non-English language, an English version will be identified, or the author contacted to check if an English version is available. If an English version is unavailable the study will be excluded. A list of excluded non-English language studies will be summarised in the appendices or supplementary material.

Studies that report only laboratory/biomechanical work, methodology for identifying new prognostic factors, or results from animal studies will be excluded. Furthermore, if multiple papers are published using the same or overlapping datasets, then only one of these papers

For peer review only

For peer review only - http://bmjopen.bmj.com/site/about/guidelines.xhtml

will be included, which will be based on the largest number of patients, the most detailed results, and/or the longest follow-up time.

Table 1: PICOTS used as selection criteria

Population	Any human population with total elbow replacement
Index prognostic factor	All possible prognostic factors
Comparator	Not applicable because all prognostic factors will be systematically identified, and the evidence will be summarised
Outcome	The outcome is failure which is defined as revision surgery. Revision surgery is defined as any secondary surgery to the prosthesis, this includes addition, removal, or alterations to all or part of the construct on the same elbow occurring at any time following the primary total elbow replacement.
Timing	Follow-up at any time after the initial TER will be evaluated (all time periods)
Setting	Any healthcare setting

Table 2: Possible prognostic factors investigated in the literature

Patient factors	Age<br>Co-morbidities or American Society of Anaesthesiologists (ASA) status<br>Ethnicity<br>Hand dominance<br>Indication for surgery<br>Sex/Gender<br>Socioeconomic status<br>Weight or body mass index (BMI)<br>Occupation<br>Activity/functional level
Implant factors	Fixation type<br>Implant brand<br>Implant design (linked/unlinked)

For peer review only

Surgery factors	Length of surgery<br>Surgical approach<br>Surgical technique (e.g. cementation technique)<br>Use of venous thromboembolism (VTE) prophylaxis<br>Use of antibiotics
Surgeons/hospital factors	Hospital experience (numbers per year)<br>Surgeon's experience (numbers per year)<br>Surgeon's grade

Search strategy

A broad search will be conducted to ensure all the relevant studies are captured in the search (high sensitivity). As the reporting of prognostic factor studies is generally poor[27], the search will aim to capture all published studies for total elbow replacement/arthroplasty (low specificity).

Electronic searches will be conducted using OVID Medline, Embase, PubMed and Cochrane Library databases. The search strategy will include a combination of subject headings (MESH) and free text searches (Supplementary File 1). The proposed search terms were developed with guidance from an experienced information scientist at the University of Manchester. The electronic search will not be limited by the date of publication or the language, or to the human population. This search will be supplemented with a screening of reference lists of the included studies and relevant review studies to identify any further studies that could have been missed in the electronic search. All duplicates will be removed using EndNote 20 (Philadelphia, USA).

Study screening

The study screening process will undergo two phases: 1) screening of titles and abstracts and 2) full-text screening. The screening process will be blinded and all studies in each phase will be screened independently by two reviewers against the specified inclusion and exclusion criteria (Table 1). ZH will screen 100% of the studies and the second independent review will be performed by CKG and LMB (50% each) (Figure 1). The screening will be conservative, and studies will be included in the next round of screening unless there is an

For peer review only

agreement to remove them. Copies of the relevant and unclear studies will be obtained and read thoroughly by the two independent reviewers. Any disagreement will be resolved by discussion or involvement of a third reviewer (JCS). The reason to exclude any study in the full-text screening phase will be provided in a supplementary file. Rayyan software (Cambridge, USA) will be used for the management of the search results.

Pilot testing of the selection process will be undertaken using 30 articles selected at random and screened by the reviewers. A meeting will be held to review the screening process to determine whether any changes need to be made.

Data extraction

The data will be extracted using a standardised tool that will be based on the Checklist for Critical Appraisal and Data Extraction for Systematic Reviews of Prediction Modelling Studies for Prognostic Factors (CHARMS-PF) [12]. CHARMS-PF covers nine domains: 1) Source of data 2) Participants 3) Outcomes to be predicted 4) Prognostic factors 5) Sample size 6) Missing data 7) Analysis 8) Results and 9) Interpretation and discussion.

The data from all included studies will be extracted by two independent reviewers, ZH will complete 100% of data extraction and the second independent data extraction will be performed by CKG and LMB (50% each), and any disagreement will be resolved by discussion. Where disagreement remains, a third reviewer (JCS) will be asked to assess the data and the majority opinion will be accepted. The data extraction tool will initially undergo pilot testing using 10% of the included studies chosen at random and a meeting will be held to review and determine whether any changes need to be made.

Risk of bias

The methodological quality or risk of bias in the included studies will be assessed using the Quality In Prognostic Studies (QUIPS) tool which has been recommended and developed specifically for prognostic factor studies[28 29]. The tool has 6 domains: 1) Selection of study participation 2) Study attrition 3) Prognostic factor measurement 4) Outcome measurement 5) Study co-variates, and 6) Statistical analysis and reporting.

Two independent reviewers, (ZH) and (TH), will assess each domain and mark with responses ‘yes’, ‘no’ or ‘unclear’. The risk of bias (ROB) in each domain will then be classified into ‘low’, ‘moderate’, or ‘high’. Any disagreement between the two reviewers will be

For peer review only - http://bmjopen.bmj.com/site/about/guidelines.xhtml

resolved by discussion. Where there is disagreement a third reviewer (JCS) will be asked to assess the study and the majority opinion will be accepted.

Data synthesis and meta-analysis

For the quantitative synthesis, prognostic estimates and their precisions will be extracted for each prognostic factor. This will include the risk ratio (RR), odds ratio (OR), or hazard ratio (HR) to measure the prognostic effect, and standard errors and confidence intervals to measure the precision. Mean difference will also be accepted to measure associations for continuous variables. Both adjusted and unadjusted prognostic effects will be considered, however, adjusted effect measures will be favoured in the interpretations[12]. For missing estimates of prognostic values, the primary author will be contacted and estimates will be requested, or, where appropriate, statistical methods described by Parmar et al (Stat Med 1998) and Tierney et al (Trials 2007) will be used to estimate unadjusted hazard ratios and their variances[30 31].

Meta-analyses for each prognostic factor will be undertaken for studies that are deemed to be sufficiently robust and comparable. Several challenges are likely to arise due to heterogeneity between studies, therefore, subgroup analysis will be undertaken of groups of studies with similar characteristics. If the number of studies allows, then separate meta-analyses will be undertaken for groups with similar prognostic effect measures, for example:

- Hazard ratios, odds ratios, and risk ratios separately
- Unadjusted and adjusted associations separately
- Prognostic factor effects at distinct cut points (or groups of similar cut points) separately
- Prognostic factor effects corresponding to a linear trend (association) separately
- Prognostic factor effects corresponding to non-linear trends separately
- Prognostic factors at different times of measurement (preoperatively, intraoperatively or postoperatively) separately
- Each method of measurement (for factors and outcomes) separately.

Sensitivity analysis will also be performed when there is heterogeneity between studies caused by differences in:

For peer review only - http://bmjopen.bmj.com/site/about/guidelines.xhtml

- The outcome (revision surgery) definition
- The participations baseline characteristics (case-mix)
- The risk of bias
- The study designs
- The type of total elbow replacement included in the study

If heterogeneity persists despite subgroup analyses, then a random effect approach will be used to allow for unexplained heterogeneity across included studies. This will be performed if 5 or more studies are included in a meta-analysis, otherwise, a fixed effect approach will be used[32].

Statistical analyses will be performed using R version 4.2.2 or later and RStudio software (Vienna, Austria). If quantitative synthesis is not possible because of issues such as a paucity of evidence, overall evidence quality and methodological heterogeneity, a narrative synthesis of the evidence will be undertaken.

Assessment of heterogeneity

Pooled data will be used in the review and heterogeneity will be assessed between each group using forest plots with 95% confidence intervals for the pooled measures. Heterogeneity will also be assessed by calculating the Q statistic and I^2. If there is heterogeneity across the included studies ($I^2 > 40\%$)[33], then the overall pooled data estimate will not be reported. The heterogeneity results will still be reported.

Reporting bias

Reporting deficiencies will be assessed using a funnel plot which addresses "small-study effects". The funnel plot will be used when 10 or more studies are examining the same prognostic factor and included in the same meta-analysis[34]. The Peters' and Debray's tests will be used to test for asymmetry in the funnel plot[34 35]. The asymmetry in the plot cannot differentiate between differences caused by bias (publication or selective reporting biases) or heterogeneity, therefore, the results will be interpreted with caution.

For peer review only - http://bmjopen.bmj.com/site/about/guidelines.xhtml

Reporting

The quality of evidence relating to each prognostic factor will be evaluated using the adapted Grades of Recommendation Assessment, Development, and Evaluation (GRADE) framework that is specific to systematic reviews of prognostic factor research [36]. The GRADE contains five domains:

- Risk of bias
- Inconsistency
- Imprecision
- Indirectness
- And publication bias

The GRADE system will be used to summarise whether the evidence for each prognostic value is of high, moderate, low, or very low quality. The online The GRADEpro Guideline Development Tool (GDT) will be used.

For peer review only

For peer review only - http://bmjopen.bmj.com/site/about/guidelines.xhtml

Author's contributors: ZH, LKF, ACW, and JCS provided the idea of the topic. ZH designed and wrote the protocol. CKG contributed to designing the search strategy, screening process, and data extraction. TH contributed to the methodology of the risk of bias assessment and group for sensitivity analysis. JCS contributed to the design of the statistical methods. ACW contributed to a list of a priori prognostic factors. ZH coordinated the whole process. All authors read, provided feedback, input into the methodology, and approved the final manuscript. LKF, ACW and JCS contributed to this work equally.

PROSPERO registration number CRD42023384756

Ethics and dissemination: Ethical approval will not be necessary, as all data are already in the public domain. Results will be published in a peer-reviewed scientific journal.

Acknowledgements: None

Competing Interests: None declared

Funding: This work was supported by funding from

- The joint National Joint Registry (NJR) and Royal College of Surgeons of England (RCS England) Research Fellowship Grant number: NA
- The John Charnley Trust Grant number: NA
- Versus Arthritis Grant number: 21755

Patient consent for publication: Not required.

Figure legends:

Figure 1: Summary of the systematic review process

For peer review only

For peer review only - http://bmjopen.bmj.com/site/about/guidelines.xhtml

REFERENCES:

1. Macken AA, Prkic A, Kodde IF, et al. Global trends in indications for total elbow arthroplasty: a systematic review of national registries. *EFORT Open Rev* 2020;5(4):215-20. doi: 10.1302/2058-5241.5.190036 [published Online First: 20200402]
2. Chou TA, Ma HH, Wang JH, et al. Total elbow arthroplasty in patients with rheumatoid arthritis. *Bone Joint J* 2020;102-b(8):967-80. doi: 10.1302/0301-620x.102b8.Bjj-2019-1465.R1
3. Evans JP, Evans JT, Mohammad HR, et al. How long does an elbow replacement last? A systematic review and meta-analysis of case-series and national registry reports with more than 10 years of follow-up. *Acta Orthopaedica* 2022;93:495-502. doi: 10.2340/17453674.2022.2753
4. Krenek L, Farng E, Zingmond D, et al. Complication and revision rates following total elbow arthroplasty. *J Hand Surg Am* 2011;36(1):68-73. doi: 10.1016/j.jhsa.2010.09.036
5. Little CP, Graham AJ, Carr AJ. Total elbow arthroplasty: a systematic review of the literature in the English language until the end of 2003. *J Bone Joint Surg Br* 2005;87(4):437-44. doi: 10.1302/0301-620x.87b4.15692
6. Prkic A, Welsink C, The B, et al. Why does total elbow arthroplasty fail today? A systematic review of recent literature. *Arch Orthop Trauma Surg* 2017;137(6):761-69. doi: 10.1007/s00402-017-2687-x [published Online First: 20170409]

For peer review only - http://bmjopen.bmj.com/site/about/guidelines.xhtml

7. Voloshin I, Schippert DW, Kakar S, et al. Complications of total elbow replacement: a systematic review. *J Shoulder Elbow Surg* 2011;20(1):158-68. doi: 10.1016/j.jse.2010.08.026

8. Welsink CL, Lambers KTA, van Deurzen DFP, et al. Total Elbow Arthroplasty: A Systematic Review. *JBJS Rev* 2017;5(7):e4. doi: 10.2106/jbjs.Rvw.16.00089

9. Chin K, Lambert S. Revision total elbow replacement. *J Clin Orthop Trauma* 2021;20:101495. doi: 10.1016/j.jcot.2021.101495 [published Online First: 20210703]

10. Wagner ER, Ransom JE, Kremers HM, et al. Comparison of the hospital costs for two-stage reimplantation for deep infection, single-stage revision and primary total elbow arthroplasty. *Shoulder Elbow* 2017;9(4):279-84. doi: 10.1177/1758573217706364 [published Online First: 20170508]

11. Riley RD, Hayden JA, Steyerberg EW, et al. Prognosis Research Strategy (PROGRESS) 2: prognostic factor research. *PLoS Med* 2013;10(2):e1001380. doi: 10.1371/journal.pmed.1001380 [published Online First: 20130205]

12. Riley RD, Moons KGM, Snell KIE, et al. A guide to systematic review and meta-analysis of prognostic factor studies. *Bmj* 2019;364:k4597. doi: 10.1136/bmj.k4597 [published Online First: 20190130]

13. Fevang BT, Lie SA, Havelin LI, et al. Results after 562 total elbow replacements: a report from the Norwegian Arthroplasty Register. *J Shoulder Elbow Surg* 2009;18(3):449-56. doi: 10.1016/j.jse.2009.02.020

14. Jenkins PJ, Watts AC, Norwood T, et al. Total elbow replacement: outcome of 1,146 arthroplasties from the Scottish Arthroplasty Project. *Acta Orthop* 2013;84(2):119-23. doi: 10.3109/17453674.2013.784658 [published Online First: 20130314]

For peer review only

15. Krukhaug Y, Hallan G, Dybvik E, et al. A survivorship study of 838 total elbow replacements: a report from the Norwegian Arthroplasty Register 1994-2016. *J Shoulder Elbow Surg* 2018;27(2):260-69. doi: 10.1016/j.jse.2017.10.018

16. Plaschke HC, Thillemann TM, Brorson S, et al. Implant survival after total elbow arthroplasty: a retrospective study of 324 procedures performed from 1980 to 2008. *J Shoulder Elbow Surg* 2014;23(6):829-36. doi: 10.1016/j.jse.2014.02.001 [published Online First: 20140422]

17. Skyttä ET, Eskelinen A, Paavolainen P, et al. Total elbow arthroplasty in rheumatoid arthritis: a population-based study from the Finnish Arthroplasty Register. *Acta Orthop* 2009;80(4):472-7. doi: 10.3109/17453670903110642

18. Viveen J, van den Bekerom MPJ, Doornberg JN, et al. Use and outcome of 1,220 primary total elbow arthroplasties from the Australian Orthopaedic Association National Joint Arthroplasty Replacement Registry 2008-2018. *Acta Orthop* 2019;90(6):511-16. doi: 10.1080/17453674.2019.1657342 [published Online First: 20190827]

19. Baghdadi YMK, Veillette CJH, Malone AA, et al. Total elbow arthroplasty in obese patients. *The Journal of bone and joint surgery American volume* 2014;96(9):e70-e70. doi: 10.2106/JBJS.M.00364

20. Riley RD, Moons KG, Hayden JA, et al. Prognostic factor research in healthcare. In: van der Windt DA, Hemingway H, Croft P, et al., eds. Prognosis Research in Healthcare: Concepts, Methods, and Impact: Oxford University Press 2019:0.

21. Damen JAAG, Hooft L. The increasing need for systematic reviews of prognosis studies: strategies to facilitate review production and improve quality of primary research. *Diagnostic and Prognostic Research* 2019;3(1):2. doi: 10.1186/s41512-019-0049-6

For peer review only

22. Moher D, Shamseer L, Clarke M, et al. Preferred reporting items for systematic review and meta-analysis protocols (PRISMA-P) 2015 statement. *Systematic Reviews* 2015;4(1):1. doi: 10.1186/2046-4053-4-1

23. Hemingway H, Croft P, Perel P, et al. Prognosis research strategy (PROGRESS) 1: a framework for researching clinical outcomes. *Bmj* 2013;346:e5595. doi: 10.1136/bmj.e5595 [published Online First: 20130205]

24. Hingorani AD, Windt DAvd, Riley RD, et al. Prognosis research strategy (PROGRESS) 4: Stratified medicine research. *BMJ : British Medical Journal* 2013;346:e5793. doi: 10.1136/bmj.e5793

25. Steyerberg EW, Moons KGM, van der Windt DA, et al. Prognosis Research Strategy (PROGRESS) 3: Prognostic Model Research. *PLOS Medicine* 2013;10(2):e1001381. doi: 10.1371/journal.pmed.1001381

26. Liebs TR, Splietker F, Hassenpflug J. Is a Revision a Revision? An Analysis of National Arthroplasty Registries' Definitions of Revision. *Clinical orthopaedics and related research* 2015;473(11):3421-30. doi: 10.1007/s11999-015-4255-4

27. Geersing GJ, Bouwmeester W, Zuithoff P, et al. Search filters for finding prognostic and diagnostic prediction studies in Medline to enhance systematic reviews. *PLoS One* 2012;7(2):e32844. doi: 10.1371/journal.pone.0032844 [published Online First: 20120229]

28. Hayden JA, Côté P, Bombardier C. Evaluation of the quality of prognosis studies in systematic reviews. *Ann Intern Med* 2006;144(6):427-37. doi: 10.7326/0003-4819-144-6-200603210-00010

For peer review only

29. Hayden JA, van der Windt DA, Cartwright JL, et al. Assessing bias in studies of prognostic factors. *Ann Intern Med* 2013;158(4):280-6. doi: 10.7326/0003-4819-158-4-201302190-00009

30. Parmar MK, Torri V, Stewart L. Extracting summary statistics to perform meta-analyses of the published literature for survival endpoints. *Stat Med* 1998;17(24):2815-34. doi: 10.1002/(sici)1097-0258(19981230)17:24<2815::aid-sim110>3.0.co;2-8

31. Tierney JF, Stewart LA, Ghersi D, et al. Practical methods for incorporating summary time-to-event data into meta-analysis. *Trials* 2007;8(1):16. doi: 10.1186/1745-6215-8-16

32. IntHout J, Ioannidis JPA, Borm GF. The Hartung-Knapp-Sidik-Jonkman method for random effects meta-analysis is straightforward and considerably outperforms the standard DerSimonian-Laird method. *BMC Medical Research Methodology* 2014;14(1):25. doi: 10.1186/1471-2288-14-25

33. Higgins JPT, Thomas J, Chandler J, et al. Cochrane Handbook for Systematic Reviews of Interventions. 2nd Edition. Chichester (UK): John Wiley & Sons, 2019. 2022.

34. Debray TPA, Moons KGM, Riley RD. Detecting small-study effects and funnel plot asymmetry in meta-analysis of survival data: A comparison of new and existing tests. *Research synthesis methods* 2018;9(1):41-50. doi: 10.1002/jrsm.1266 [published Online First: 2017/11/28]

35. Peters JL, Sutton AJ, Jones DR, et al. Comparison of two methods to detect publication bias in meta-analysis. *Jama* 2006;295(6):676-80. doi: 10.1001/jama.295.6.676

36. Huguet A, Hayden JA, Stinson J, et al. Judging the quality of evidence in reviews of prognostic factor research: adapting the GRADE framework. *Syst Rev* 2013;2:71. doi: 10.1186/2046-4053-2-71 [published Online First: 20130905]

For peer review only

For peer review only

For peer review only - http://bmjopen.bmj.com/site/about/guidelines.xhtml

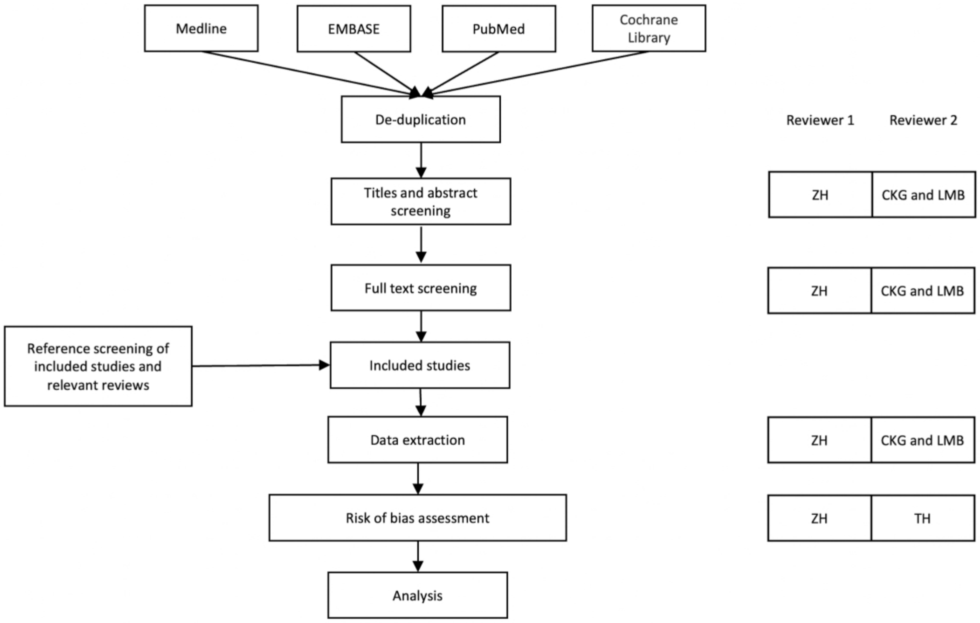

Figure 1: Summary of the systematic review process

394x253mm (300 x 300 DPI)

Supplementary File 1

Electronic search strategies

MEDLINE search - Ovid interface

#	Query
1	exp Arthroplasty, Replacement, Elbow/
2	total elbow replac*.mp. [mp=title, book title, abstract, original title, name of substance word, subject heading word, floating sub-heading word, keyword heading word, organism supplementary concept word, protocol supplementary concept word, rare disease supplementary concept word, unique identifier, synonyms]
3	total elbow arthropl*.mp. [mp=title, book title, abstract, original title, name of substance word, subject heading word, floating sub-heading word, keyword heading word, organism supplementary concept word, protocol supplementary concept word, rare disease supplementary concept word, unique identifier, synonyms]
4	(total and elbow and replac*).ab.
5	(total and elbow and replac*).ti.
6	(total and elbow and arthropl*).ab.
7	(total and elbow and arthropl*).ti.
8	((elbow adj2 arthroplast*) or (elbow adj2 replac*)).mp.
9	1 or 2 or 3 or 4 or 5 or 6 or 7 or 8

EMBASE search- Ovid interface

#	Query
1	exp elbow replacement/ or exp elbow arthroplasty/
2	total elbow replac*.mp. [mp=title, abstract, heading word, drug trade name, original title, device manufacturer, drug manufacturer, device trade name, keyword heading word, floating subheading word, candidate term word]
3	total elbow arthropl*.mp. [mp=title, abstract, heading word, drug trade name, original title, device manufacturer, drug manufacturer, device trade name, keyword heading word, floating subheading word, candidate term word]

For peer review only

4	(total and elbow and replac*).ab.
5	(total and elbow and replac*).ti.
6	(total and elbow and arthropl*).ab.
7	(total and elbow and arthropl*).ti.
8	((elbow adj2 arthroplast*) or (elbow adj2 replac*)).mp.
9	1 or 2 or 3 or 4 or 5 or 6 or 7 or 8
10	limit 9 to conference abstract status
11	9 not 10

PubMed search

ID	Search
#1	"Arthroplasty, Replacement, Elbow"[Mesh]
#2	total elbow replac*<br><br>("total"[All Fields] OR "totaled"[All Fields] OR "totaling"[All Fields] OR "totalled"[All Fields] OR "totalling"[All Fields] OR "totals"[All Fields]) AND ("elbow"[MeSH Terms] OR "elbow"[All Fields] OR "elbow joint"[MeSH Terms] OR ("elbow"[All Fields] AND "joint"[All Fields]) OR "elbow joint"[All Fields] OR "elbow s"[All Fields] OR "elbows"[All Fields]) AND "arthropl*"[All Fields]
#3	total elbow arthropl*<br><br>("total"[All Fields] OR "totaled"[All Fields] OR "totaling"[All Fields] OR "totalled"[All Fields] OR "totalling"[All Fields] OR "totals"[All Fields]) AND ("elbow"[MeSH Terms] OR "elbow"[All Fields] OR "elbow joint"[MeSH Terms] OR ("elbow"[All Fields] AND "joint"[All Fields]) OR "elbow joint"[All Fields] OR "elbow s"[All Fields] OR "elbows"[All Fields]) AND "replac*"[All Fields]
#4	"total"[Title/Abstract] AND "elbow"[Title/Abstract] AND "replac*"[Title/Abstract]
#5	"total"[Title/Abstract] AND "elbow"[Title/Abstract] AND "arthropl*"[Title/Abstract]
#6	(elbow n2 arthropl*) OR (elbow n2 replac*)<br><br>(("elbow"[MeSH Terms] OR "elbow"[All Fields] OR "elbow joint"[MeSH Terms] OR ("elbow"[All Fields] AND "joint"[All Fields]) OR "elbow joint"[All Fields] OR "elbow s"[All Fields] OR "elbows"[All Fields]) AND "n2"[All Fields] AND "arthropl*"[All Fields]) OR (("elbow"[MeSH Terms] OR "elbow"[All Fields] OR "elbow joint"[MeSH Terms] OR ("elbow"[All Fields] AND "joint"[All Fields]) OR "elbow joint"[All Fields] OR "elbow s"[All Fields] OR "elbows"[All Fields]) AND "n2"[All Fields] AND "replac*"[All Fields])
#9	1 or 2 or 3 or 4 or 5 or 6

For peer review only

Cochrane library search

ID	Search
#1	MeSH descriptor: [Arthroplasty, Replacement, Elbow] explode all trees
#2	total elbow replac*
#3	total elbow arthropl*
#4	(Total) AND (Elbow) AND ("replac*"or"arthropl*") (Word variations have been searched)
#5	#1 OR #2 OR #3 OR #4

For peer review only

PRISMA 2020 Checklist

Section and Topic	Item #	Checklist item	Location where item is reported
**TITLE**			
Title	1	Identify the report as a systematic review.	Title page
**ABSTRACT**			
Abstract	2	See the PRISMA 2020 for Abstracts checklist.	Abstract
**INTRODUCTION**			
Rationale	3	Describe the rationale for the review in the context of existing knowledge.	Introduction
Objectives	4	Provide an explicit statement of the objective(s) or question(s) the review addresses.	Introduction
**METHODS**			
Eligibility criteria	5	Specify the inclusion and exclusion criteria for the review and how studies were grouped for the syntheses.	Methods
Information sources	6	Specify all databases, registers, websites, organisations, reference lists and other sources searched or consulted to identify studies. Specify the date when each source was last searched or consulted.	Methods
Search strategy	7	Present the full search strategies for all databases, registers and websites, including any filters and limits used.	Methods
Selection process	8	Specify the methods used to decide whether a study met the inclusion criteria of the review, including how many reviewers screened each record and each report retrieved, whether they worked independently, and if applicable, details of automation tools used in the process.	Methods
Data collection process	9	Specify the methods used to collect data from reports, including how many reviewers collected data from each report, whether they worked independently, any processes for obtaining or confirming data from study investigators, and if applicable, details of automation tools used in the process.	Methods
Data items	10a	List and define all outcomes for which data were sought. Specify whether all results that were compatible with each outcome domain in each study were sought (e.g. for all measures, time points, analyses), and if not, the methods used to decide which results to collect.	Methods
	10b	List and define all other variables for which data were sought (e.g. participant and intervention characteristics, funding sources). Describe any assumptions made about any missing or unclear information.	Methods
Study risk of bias assessment	11	Specify the methods used to assess risk of bias in the included studies, including details of the tool(s) used, how many reviewers assessed each study and whether they worked independently, and if applicable, details of automation tools used in the process.	Methods
Effect measures	12	Specify for each outcome the effect measure(s) (e.g. risk ratio, mean difference) used in the synthesis or presentation of results.	Methods
Synthesis methods	13a	Describe the processes used to decide which studies were eligible for each synthesis (e.g. tabulating the study intervention characteristics and comparing against the planned groups for each synthesis (item #5)).	Methods
	13b	Describe any methods required to prepare the data for presentation or synthesis, such as handling of missing summary statistics, or data conversions.	Methods
	13c	Describe any methods used to tabulate or visually display results of individual studies and syntheses.	Methods
	13d	Describe any methods used to synthesize results and provide a rationale for the choice(s). If meta-analysis was performed, describe the model(s), method(s) to identify the presence and extent of statistical heterogeneity, and software package(s) used.	Methods
	13e	Describe any methods used to explore possible causes of heterogeneity among study results (e.g. subgroup analysis, meta-regression).	Methods
	13f	Describe any sensitivity analyses conducted to assess robustness of the synthesized results.	Methods
Reporting bias assessment	14	Describe any methods used to assess risk of bias due to missing results in a synthesis (arising from reporting biases).	Methods
Certainty	15	Describe any methods used to assess certainty (or confidence) in the body of evidence for an outcome.	Methods

For peer review only

PRISMA 2020 Checklist

Section and Topic	Item #	Checklist item	Location where item is reported
assessment			
**RESULTS**			
Study selection	16a	Describe the results of the search and selection process, from the number of records identified in the search to the number of studies included in the review, ideally using a flow diagram.	Not applicable
	16b	Cite studies that might appear to meet the inclusion criteria, but which were excluded, and explain why they were excluded.	Not applicable
Study characteristics	17	Cite each included study and present its characteristics.	Not applicable
Risk of bias in studies	18	Present assessments of risk of bias for each included study.	Not applicable
Results of individual studies	19	For all outcomes, present, for each study: (a) summary statistics for each group (where appropriate) and (b) an effect estimate and its precision (e.g. confidence/credible interval), ideally using structured tables or plots.	Not applicable
Results of syntheses	20a	For each synthesis, briefly summarise the characteristics and risk of bias among contributing studies.	Not applicable
	20b	Present results of all statistical syntheses conducted. If meta-analysis was done, present for each the summary estimate and its precision (e.g. confidence/credible interval) and measures of statistical heterogeneity. If comparing groups, describe the direction of the effect.	Not applicable
	20c	Present results of all investigations of possible causes of heterogeneity among study results.	Not applicable
	20d	Present results of all sensitivity analyses conducted to assess the robustness of the synthesized results.	Not applicable
Reporting biases	21	Present assessments of risk of bias due to missing results (arising from reporting biases) for each synthesis assessed.	Not applicable
Certainty of evidence	22	Present assessments of certainty (or confidence) in the body of evidence for each outcome assessed.	Not applicable
**DISCUSSION**			
Discussion	23a	Provide a general interpretation of the results in the context of other evidence.	Not applicable
	23b	Discuss any limitations of the evidence included in the review.	Not applicable
	23c	Discuss any limitations of the review processes used.	Not applicable
	23d	Discuss implications of the results for practice, policy, and future research.	Not applicable
**OTHER INFORMATION**			
Registration and protocol	24a	Provide registration information for the review, including register name and registration number, or state that the review was not registered.	Completed
	24b	Indicate where the review protocol can be accessed, or state that a protocol was not prepared.	Completed
	24c	Describe and explain any amendments to information provided at registration or in the protocol.	None

For peer review only

For peer review only - http://bmjopen.bmj.com/site/about/guidelines.xhtml

PRISMA 2020 Checklist

Section and Topic	Item #	Checklist item	Location where item is reported
Support	25	Describe sources of financial or non-financial support for the review, and the role of the funders or sponsors in the review.	Completed
Competing interests	26	Declare any competing interests of review authors.	Completed
Availability of data, code and other materials	27	Report which of the following are publicly available and where they can be found: template data collection forms; data extracted from included studies; data used for all analyses; analytic code; any other materials used in the review.	Not applicable

From: Page MJ, McKenzie JE, Bossuyt PM, Boutron I, Hoffmann TC, Mulrow CD, et al. The PRISMA 2020 statement: an updated guideline for reporting systematic reviews. BMJ 2021;372:n71. doi: 10.1136/bmj.n71

For more information, visit: http://www.prisma-statement.org/

For peer review only

BMJ Open

Prognostic factors associated with failure of total elbow replacement: a protocol for a systematic review

Journal:	*BMJ Open*
Manuscript ID	bmjopen-2023-071705.R1
Article Type:	Protocol
Date Submitted by the Author:	05-Jun-2023
Complete List of Authors:	Hamoodi, Zaid; The University of Manchester Centre for Epidemiology Versus Arthritis, Centre for Musculoskeletal Research; Wrightington Wigan and Leigh NHS Foundation Trust, Upper Limb Unit<br>Gehringer, Celina; The University of Manchester Centre for Epidemiology Versus Arthritis, Centre for Musculoskeletal Research, University of Manchester<br>Bull, Lucy; Health Navigator Ltd<br>Hughes, Tom; Manchester Metropolitan University Department of Health Professions; Manchester United Ltd<br>Kearsley-Fleet, Lianne ; The University of Manchester Centre for Epidemiology Versus Arthritis, Centre for Musculoskeletal Research, The University of Manchester<br>Sergeant, Jamie; The University of Manchester Centre for Epidemiology Versus Arthritis, Centre for Musculoskeletal Research, Manchester Academic Health Science Centre,; The University of Manchester, Centre for Biostatistics, School of Health Sciences, Faculty of Biology, Medicine and Health, Manchester Academic Health Science Centre<br>Watts, Adam; Wrightington Wigan and Leigh NHS Foundation Trust, Upper Limb Unit
**Primary Subject Heading**:	Surgery
Secondary Subject Heading:	Epidemiology
Keywords:	ORTHOPAEDIC & TRAUMA SURGERY, Adult orthopaedics < ORTHOPAEDIC & TRAUMA SURGERY, Elbow & shoulder < ORTHOPAEDIC & TRAUMA SURGERY, Musculoskeletal disorders < ORTHOPAEDIC & TRAUMA SURGERY

SCHOLARONE™
Manuscripts

Title page

Title of the article:

Prognostic factors associated with failure of total elbow replacement: a protocol for a systematic review

Full name, postal address and e-mail of the corresponding author:

Zaid Hamoodi

Office 2.612, Second floor, Stopford building, Centre for Epidemiology Versus Arthritis, Division of Musculoskeletal and Dermatological Sciences, University of Manchester, UK

Zaid.hamoodi-2@postgrad.manchester.ac.uk

Full name, department, institution, city and country of all co-authors

- Mr Zaid Hamoodi. 1) Centre for Epidemiology Versus Arthritis, Centre for Musculoskeletal Research, University of Manchester, Manchester Academic Health Science Centre, Manchester, UK 2) Upper Limb Unit, Wrightington Hospital, Wrightington, Wigan and Leigh Teaching Hospitals NHS Foundation Trust, Wigan, UK
- Miss Celina K Gehringer. Centre for Epidemiology Versus Arthritis, Centre for Musculoskeletal Research, University of Manchester, Manchester Academic Health Science Centre, Manchester, UK Miss Lucy M Bull. Health Navigator Ltd, London, UK
- Miss Lucy M Bull. Health Navigator Ltd, London, Greater London, UK
- Dr Tom Hughes. 1) Department of Health Professions, Manchester Metropolitan University, Manchester, UK 2) Manchester United Ltd, Manchester, UK
- Dr Lianne Kearsley-Fleet. Centre for Epidemiology Versus Arthritis, Centre for Musculoskeletal Research, University of Manchester, Manchester Academic Health Science Centre, Manchester, UK
- Dr Jamie C Sergeant. 1) Centre for Biostatistics, School of Health Sciences, Faculty of Biology, Medicine and Health, University of Manchester, Manchester Academic Health Science Centre, Manchester, UK 2) Centre for Epidemiology Versus Arthritis, Centre for Musculoskeletal Research, University of Manchester, Manchester Academic Health Science Centre, Manchester, UK

For peer review only

- Professor Adam C Watts. Upper Limb Unit, Wrightington Hospital, Wrightington, Wigan and Leigh Teaching Hospitals NHS Foundation Trust, Wigan, UK

Word count, excluding title page, abstract, references, figures and tables

2617

Keywords

Arthroplasty, Replacement, Elbow, Prognosis, Prognostic factors

For peer review only

Abstract

Introduction

Total elbow replacement (TER) has higher failure rates requiring revision surgery compared with the replacement of other joints. Understanding the factors associated with failure is essential for informed decision-making between patients and clinicians, and for reducing the failure rate. This review aims to identify, describe, and appraise the literature examining prognostic factors for failure of TER.

Methods and analysis

This systematic review will be conducted and reported in line with the PRISMA-P guidelines. Electronic literature searches will be conducted using Medline, EMBASE, PubMed, and Cochrane. The search strategy will be broad, including a combination of subject headings (MESH) and free text search. This search will be supplemented with a screening of reference lists of the included studies and relevant reviews. Two independent reviewers will screen all search results in two stages (title and abstract, and full text) based on the PICOTS criteria. The types of evidence included will be randomised trials, non-randomised trials, prospective and retrospective cohort studies, registry studies, and case-control studies. If the literature lacks enough studies, then case series with 50 or more TERs will be considered for inclusion. Data extraction and risk of bias assessment for included studies will be performed by two independent reviewers using the CHARMS-PF and QUIPS tools.

Meta-analyses of prognostic estimates for each factor will be undertaken for studies that are deemed to be sufficiently robust and comparable. Several challenges are likely to arise due to heterogeneity between studies, therefore, subgroup and sensitivity analyses will be performed to account for the differences between studies. Heterogeneity will be assessed using Q and I^2 statistics. If I^2 >40% then pooled estimates will not be reported. When quantitative synthesis is not possible, a narrative synthesis will be undertaken. The quality of the evidence for each prognostic factor will be assessed using the GRADE tool.

For peer review only

For peer review only - http://bmjopen.bmj.com/site/about/guidelines.xhtml

Strengths and limitations of this study

- This is the first systematic review to focus on identifying and appraising studies evaluating prognostic factors associated with failure of total elbow replacement.
- This review will follow the methodological advances in performing systematic reviews and meta-analyses in prognosis research proposed by the PROGnosis REsearch Strategy (PROGRESS) and The Cochrane Prognosis Methods Group (PMG).
- This review will use the recommended checklists and tools including the Patient, Intervention, Comparison, Outcome, Time, and Setting (PICOTS) model for study selection, the Checklist for Critical Appraisal and Data Extraction for Systematic Reviews of Prediction Modelling Studies for Prognostic Factors (CHARMS-PF) for data extraction, and the Quality In Prognostic Studies (QUIPS) tool to assess the risk of bias.
- This review may be limited by the quality of and the heterogeneity between the included studies. We aim to address this by performing statistical analysis of heterogeneity combined with subgroup and sensitivity analyses.

For peer review only

INTRODUCTION

Total elbow replacement (TER) is a recognised treatment for painful arthritic elbow and whilst rheumatoid arthritis remains the main indication, it is increasingly performed for acute trauma and post-traumatic arthritis[1]. Despite technological advances, TER has higher failure rates than other total joint replacements, with a reported 5-year failure rate of 6-10% and 10-year failure rate of 15-19% compared to a 3% 5-year failure rate and <5% 10-year failure rate in total hip replacement [1-8]. In the event of TER failure, surgical revision is usually required but this procedure carries a risk of adverse events such as ongoing pain, disruption of the elbow extensor mechanism, reduction of bone stock, infection, ulnar nerve injury, and poor function[9]. Understanding the factors associated with failure is essential to inform the development of strategies designed to reduce failure risk and the need for subsequent revision surgery, which is a burden for both patients and society[10].

A prognostic factor is defined as any variable that is associated with a risk of a health outcome among people with a particular health condition, in this context the outcome is failure, and the condition is patients with TER[11 12]. Several existing studies have examined possible factors that may be associated with the risk of failure in TER, including primary pathology, prosthesis type, surgeon's experience, implant fixation, and whether surgery is performed in a specialised centre[2 13-19]. However, some of the findings have been inconsistent. For example, some studies examining the association between the underlying pathology and TER failure found rheumatoid arthritis to be associated with a worse prognosis while others found post-traumatic arthritis to be associated with a worse prognosis[6 16].

To the best of our knowledge, there are no systematic reviews available that have appraised, pooled, and synthesised all existing studies relating to prognostic factors for TER failure, which means that the quality of the current evidence base is unknown. This also means that it is unclear whether potential factors have been derived from single exploratory studies (thus having provisional prognostic value) or replicated and confirmed in multiple studies (thus demonstrating robust prognostic value).

For peer review only - http://bmjopen.bmj.com/site/about/guidelines.xhtml

Therefore, the purpose of this systematic review is to identify, describe, appraise, and synthesise all the current literature examining the prognostic factors associated with failure of TER. A better understanding of prognostic factors may help to explain the differences in risk of failure between patients[20], which may facilitate decisions about whether or not to proceed with TER as a definitive management option. An improved understanding of prognostic factors may also help reduce the number of potential TER revisions by improving treatment selection[21] and may help to identify areas for novel interventions[11]. This review will also identify gaps in the evidence base to aid the planning of future research and could potentially pave the way to developing a prognostic model in TER that could be used to make individualised patient predictions.

METHODS AND ANALYSIS

A systematic review will be conducted and reported in line with the Preferred Reporting Items for Systematic reviews and Meta-Analyses (PRISMA-P) guidelines[22]. It has been registered at the International prospective register of systematic reviews (PROSPERO) and will follow the published guidance and recommendation produced by the PROGnosis REsearch Strategy (PROGRESS) and The Cochrane Prognosis Methods Group (PMG) on how to undertake a systematic review and meta-analysis in prognostic factors research[11 12 20 23-25].

Patients and Public Involvement

The study aim was co-designed with the Wrightington Hospital Patients and Public Involvement (PPI) group, including members with long-term health conditions and experience of joint replacement surgery. The group also contributed to a list of possible prognostic factors to be included in the review and their ongoing involvement will help ensure that the review is relevant to patients.

Study eligibility

All peer-reviewed studies will be included if prognostic factors are investigated in patients who have undergone a primary TER, and if the selection criteria in Table 1 are met (based on the Patient, Intervention, Comparison, Outcome, Time, and Setting (PICOTS) model)[12]. Studies must include a human population and provide a prognostic estimate evaluating

For peer review only - http://bmjopen.bmj.com/site/about/guidelines.xhtml

prognostic factors for failure of total elbow replacement. This initial list of candidate prognostic factors to be considered are listed in Table 2 but, importantly, this is not exhaustive and studies will also be included if other candidate prognostic factors have been investigated. Although the primary interest is in prognostic factors measured before surgery, we will not exclude prognostic factors measured intraoperatively or postoperatively.

The outcome of failure will be defined as revision surgery. The definition of revision surgery varies between joint registries and is likely to vary between other non-registry studies[26], therefore, a broad definition for revision surgery will be used which is any secondary surgery to the prosthesis, this includes addition, removal, or alterations to all or part of the construct on the same elbow occurring at any time following the primary total elbow replacement. This may lead to the inclusion of studies that evaluate non-revision secondary surgeries as part of a composite outcome. If this occurs, these studies will undergo subgroup/sensitivity analyses.

The types of evidence included will be randomised trials, non-randomised trials, prospective and retrospective cohort studies, registry studies, and case-control studies. If the literature is lacking in studies of these types, then a case series with 50 TERs or more will be considered for inclusion. Results from case series will be interpreted with caution due to the higher risk of bias, especially selection bias. Review articles, surveys, case reports, and conference abstracts will be excluded.

If the literature is published in a non-English language, an English version will be identified, or the author contacted to check if an English version is available. If an English version is unavailable the study will be excluded. A list of excluded non-English language studies will be summarised in the appendices or supplementary material.

Studies that report only laboratory/biomechanical work, methodology for identifying new prognostic factors, or results from animal studies will be excluded. Furthermore, if multiple papers are published using the same or overlapping datasets, then only one of these papers

For peer review only

will be included, which will be based on the largest number of patients, the most detailed results, and/or the longest follow-up time.

Table 1: PICOTS used as selection criteria

Population	Any human population with total elbow replacement
Index prognostic factor	All possible prognostic factors
Comparator	Not applicable because all prognostic factors will be systematically identified, and the evidence will be summarised
Outcome	The outcome is failure which is defined as revision surgery. Revision surgery is defined as any secondary surgery to the prosthesis, this includes addition, removal, or alterations to all or part of the construct on the same elbow occurring at any time following the primary total elbow replacement.
Timing	Follow-up at any time after the initial TER will be evaluated (all time periods)
Setting	Any healthcare setting

Table 2: Possible prognostic factors investigated in the literature

Patient factors	Age<br>Co-morbidities or American Society of Anaesthesiologists (ASA) status<br>Ethnicity<br>Hand dominance<br>Indication for surgery<br>Sex/Gender<br>Socioeconomic status<br>Weight or body mass index (BMI)<br>Occupation<br>Activity/functional level
Implant factors	Fixation type<br>Implant brand<br>Implant design (linked/unlinked)

For peer review only

For peer review only - http://bmjopen.bmj.com/site/about/guidelines.xhtml

Surgery factors	Length of surgery<br>Surgical approach<br>Surgical technique (e.g. cementation technique)<br>Use of venous thromboembolism (VTE) prophylaxis<br>Use of antibiotics
Surgeons/hospital factors	Hospital experience (numbers per year)<br>Surgeon's experience (numbers per year)<br>Surgeon's grade

Search strategy

A broad search will be conducted to ensure all the relevant studies are captured in the search (high sensitivity). As the reporting of prognostic factor studies is generally poor[27], the search will aim to capture all published studies for total elbow replacement/arthroplasty (low specificity).

Electronic searches will be conducted using OVID Medline, Embase, PubMed and Cochrane Library databases. The search strategy will include a combination of subject headings (MESH) and free text searches (Supplementary File 1). The proposed search terms were developed with guidance from an experienced information scientist at the University of Manchester. The electronic search will not be limited by the date of publication or the language, or to the human population. This search will be supplemented with a screening of reference lists of the included studies and relevant review studies to identify any further studies that could have been missed in the electronic search. All duplicates will be removed using EndNote 20 (Philadelphia, USA).

Study screening

The study screening process will undergo two phases: 1) screening of titles and abstracts and 2) full-text screening. The screening process will be blinded and all studies in each phase will be screened independently by two reviewers against the specified inclusion and exclusion criteria (Table 1). ZH will screen 100% of the studies and the second independent review will be performed by CKG and LMB (50% each) (Figure 1). The screening will be conservative, and studies will be included in the next round of screening unless there is an

For peer review only

agreement to remove them. Copies of the relevant and unclear studies will be obtained and read thoroughly by the two independent reviewers. Any disagreement will be resolved by discussion or involvement of a third reviewer (JCS). The reason to exclude any study in the full-text screening phase will be provided in a supplementary file. Rayyan software (Cambridge, USA) will be used for the management of the search results.

Pilot testing of the selection process will be undertaken using 30 articles selected at random and screened by the reviewers. A meeting will be held to review the screening process to determine whether any changes need to be made.

Data extraction

The data will be extracted using a standardised tool that will be based on the Checklist for Critical Appraisal and Data Extraction for Systematic Reviews of Prediction Modelling Studies for Prognostic Factors (CHARMS-PF) [12]. CHARMS-PF covers nine domains: 1) Source of data 2) Participants 3) Outcomes to be predicted 4) Prognostic factors 5) Sample size 6) Missing data 7) Analysis 8) Results and 9) Interpretation and discussion.

The data from all included studies will be extracted by two independent reviewers, ZH will complete 100% of data extraction and the second independent data extraction will be performed by CKG and LMB (50% each), and any disagreement will be resolved by discussion. Where disagreement remains, a third reviewer (JCS) will be asked to assess the data and the majority opinion will be accepted. The data extraction tool will initially undergo pilot testing using 10% of the included studies chosen at random and a meeting will be held to review and determine whether any changes need to be made.

Risk of bias

The methodological quality or risk of bias in the included studies will be assessed using the Quality In Prognostic Studies (QUIPS) tool which has been recommended and developed specifically for prognostic factor studies[28 29]. The tool has 6 domains: 1) Selection of study participation 2) Study attrition 3) Prognostic factor measurement 4) Outcome measurement 5) Study co-variates, and 6) Statistical analysis and reporting.

Two independent reviewers, (ZH) and (TH), will assess each domain and mark with responses ‘yes’, ‘no’ or ‘unclear’. The risk of bias (ROB) in each domain will then be classified into ‘low’, ‘moderate’, or ‘high’. Any disagreement between the two reviewers will be

For peer review only

resolved by discussion. Where there is disagreement a third reviewer (JCS) will be asked to assess the study and the majority opinion will be accepted.

Data synthesis and meta-analysis

For the quantitative synthesis, prognostic estimates and their precisions will be extracted for each prognostic factor. This will include the risk ratio (RR), odds ratio (OR), or hazard ratio (HR) to measure the prognostic effect, and standard errors and confidence intervals to measure the precision. Mean difference will also be accepted to measure associations for continuous variables. Both adjusted and unadjusted prognostic effects will be considered, however, adjusted effect measures will be favoured in the interpretations[12].

Meta-analyses for each prognostic factor will be undertaken for studies that are deemed to be sufficiently robust and comparable. Several challenges are likely to arise due to heterogeneity between studies, therefore, subgroup analysis will be undertaken of groups of studies with similar characteristics. If the number of studies allows, then separate meta-analyses will be undertaken for groups with similar prognostic effect measures, for example:

- Hazard ratios, odds ratios, and risk ratios separately
- Unadjusted and adjusted associations separately
- Prognostic factor effects at distinct cut points (or groups of similar cut points) separately
- Prognostic factor effects corresponding to a linear trend (association) separately
- Prognostic factor effects corresponding to non-linear trends separately
- Prognostic factors at different times of measurement (preoperatively, intraoperatively or postoperatively) separately
- Each method of measurement (for factors and outcomes) separately.

Sensitivity analysis will also be performed when there is heterogeneity between studies caused by differences in:

- The outcome (revision surgery) definition
- The participations baseline characteristics (case-mix)
- The risk of bias

For peer review only

For peer review only - http://bmjopen.bmj.com/site/about/guidelines.xhtml

- The study designs
- The type of total elbow replacement included in the study

If heterogeneity persists despite subgroup analyses, then a random effect approach will be used to allow for unexplained heterogeneity across included studies. This will be performed if 5 or more studies are included in a meta-analysis, otherwise, a fixed effect approach will be used[30].

Statistical analyses will be performed using R version 4.2.2 or later and RStudio software (Vienna, Austria). If quantitative synthesis is not possible because of issues such as a paucity of evidence, overall evidence quality and methodological heterogeneity, a narrative synthesis of the evidence will be undertaken.

Assessment of heterogeneity

Pooled data will be used in the review and heterogeneity will be assessed between each group using forest plots with 95% confidence intervals for the pooled measures. Heterogeneity will also be assessed by calculating the Q statistic and I^2. If there is heterogeneity across the included studies ($I^2 > 40\%$)[31], then the overall pooled data estimate will not be reported. The heterogeneity results will still be reported.

Reporting bias

Reporting deficiencies will be assessed using a funnel plot which addresses "small-study effects". The funnel plot will be used when 10 or more studies are examining the same prognostic factor and included in the same meta-analysis[32]. The Peters' and Debray's tests will be used to test for asymmetry in the funnel plot[32 33]. The asymmetry in the plot cannot differentiate between differences caused by bias (publication or selective reporting biases) or heterogeneity, therefore, the results will be interpreted with caution.

Reporting

The quality of evidence relating to each prognostic factor will be evaluated using the adapted Grades of Recommendation Assessment, Development, and Evaluation (GRADE)

For peer review only - http://bmjopen.bmj.com/site/about/guidelines.xhtml

framework that is specific to systematic reviews of prognostic factor research [34]. The GRADE contains five domains:

- Risk of bias
- Inconsistency
- Imprecision
- Indirectness
- And publication bias

The GRADE system will be used to summarise whether the evidence for each prognostic value is of high, moderate, low, or very low quality. The online The GRADEpro Guideline Development Tool (GDT) will be used.

For peer review only

Author's contributors: ZH, LKF, ACW, and JCS provided the idea of the topic. ZH designed and wrote the protocol. CKG and LMB contributed to designing the search strategy, screening process, and data extraction. TH contributed to the methodology of the risk of bias assessment and group for sensitivity analysis. JCS contributed to the design of the statistical methods. ACW contributed to a list of a priori prognostic factors. ZH coordinated the whole process. All authors read, provided feedback, input into the methodology, and approved the final manuscript. LKF, ACW and JCS contributed to this work equally.

PROSPERO registration number CRD42023384756

Ethics and dissemination: Ethical approval will not be necessary, as all data are already in the public domain. Results will be published in a peer-reviewed scientific journal.

Acknowledgements: None

Competing Interests: None declared

Funding: This work was supported by funding from. ZH fellowship is funded by the NJR/RCSEng and John Charnley Trust.

- The joint National Joint Registry (NJR) and Royal College of Surgeons of England (RCS England) Research Fellowship   Grant number: NA
- The John Charnley Trust   Grant number: NA
- Versus Arthritis   Grant number: 21755

Patient consent for publication: Not required.

Data availability statement: All data relevant to the study/protocol are included in the article or uploaded as supplementary information.

Figure legends:

Figure 1: Summary of the systematic review process

For peer review only

REFERENCES:

1. Macken AA, Prkic A, Kodde IF, et al. Global trends in indications for total elbow arthroplasty: a systematic review of national registries. *EFORT Open Rev* 2020;5(4):215-20. doi: 10.1302/2058-5241.5.190036 [published Online First: 20200402]

2. Chou TA, Ma HH, Wang JH, et al. Total elbow arthroplasty in patients with rheumatoid arthritis. *Bone Joint J* 2020;102-b(8):967-80. doi: 10.1302/0301-620x.102b8.Bjj-2019-1465.R1

3. Evans JP, Evans JT, Mohammad HR, et al. How long does an elbow replacement last? A systematic review and meta-analysis of case-series and national registry reports with more than 10 years of follow-up. *Acta Orthopaedica* 2022;93:495-502. doi: 10.2340/17453674.2022.2753

4. Krenek L, Farng E, Zingmond D, et al. Complication and revision rates following total elbow arthroplasty. *J Hand Surg Am* 2011;36(1):68-73. doi: 10.1016/j.jhsa.2010.09.036

5. Little CP, Graham AJ, Carr AJ. Total elbow arthroplasty: a systematic review of the literature in the English language until the end of 2003. *J Bone Joint Surg Br* 2005;87(4):437-44. doi: 10.1302/0301-620x.87b4.15692

6. Prkic A, Welsink C, The B, et al. Why does total elbow arthroplasty fail today? A systematic review of recent literature. *Arch Orthop Trauma Surg* 2017;137(6):761-69. doi: 10.1007/s00402-017-2687-x [published Online First: 20170409]

7. Voloshin I, Schippert DW, Kakar S, et al. Complications of total elbow replacement: a systematic review. *J Shoulder Elbow Surg* 2011;20(1):158-68. doi: 10.1016/j.jse.2010.08.026

8. Welsink CL, Lambers KTA, van Deurzen DFP, et al. Total Elbow Arthroplasty: A Systematic Review. *JBJS Rev* 2017;5(7):e4. doi: 10.2106/jbjs.Rvw.16.00089

9. Chin K, Lambert S. Revision total elbow replacement. *J Clin Orthop Trauma* 2021;20:101495. doi: 10.1016/j.jcot.2021.101495 [published Online First: 20210703]

For peer review only

For peer review only - http://bmjopen.bmj.com/site/about/guidelines.xhtml

10. Wagner ER, Ransom JE, Kremers HM, et al. Comparison of the hospital costs for two-stage reimplantation for deep infection, single-stage revision and primary total elbow arthroplasty. *Shoulder Elbow* 2017;9(4):279-84. doi: 10.1177/1758573217706364 [published Online First: 20170508]

11. Riley RD, Hayden JA, Steyerberg EW, et al. Prognosis Research Strategy (PROGRESS) 2: prognostic factor research. *PLoS Med* 2013;10(2):e1001380. doi: 10.1371/journal.pmed.1001380 [published Online First: 20130205]

12. Riley RD, Moons KGM, Snell KIE, et al. A guide to systematic review and meta-analysis of prognostic factor studies. *Bmj* 2019;364:k4597. doi: 10.1136/bmj.k4597 [published Online First: 20190130]

13. Fevang BT, Lie SA, Havelin LI, et al. Results after 562 total elbow replacements: a report from the Norwegian Arthroplasty Register. *J Shoulder Elbow Surg* 2009;18(3):449-56. doi: 10.1016/j.jse.2009.02.020

14. Jenkins PJ, Watts AC, Norwood T, et al. Total elbow replacement: outcome of 1,146 arthroplasties from the Scottish Arthroplasty Project. *Acta Orthop* 2013;84(2):119-23. doi: 10.3109/17453674.2013.784658 [published Online First: 20130314]

15. Krukhaug Y, Hallan G, Dybvik E, et al. A survivorship study of 838 total elbow replacements: a report from the Norwegian Arthroplasty Register 1994-2016. *J Shoulder Elbow Surg* 2018;27(2):260-69. doi: 10.1016/j.jse.2017.10.018

16. Plaschke HC, Thillemann TM, Brorson S, et al. Implant survival after total elbow arthroplasty: a retrospective study of 324 procedures performed from 1980 to 2008. *J Shoulder Elbow Surg* 2014;23(6):829-36. doi: 10.1016/j.jse.2014.02.001 [published Online First: 20140422]

17. Skyttä ET, Eskelinen A, Paavolainen P, et al. Total elbow arthroplasty in rheumatoid arthritis: a population-based study from the Finnish Arthroplasty Register. *Acta Orthop* 2009;80(4):472-7. doi: 10.3109/17453670903110642

For peer review only

For peer review only - http://bmjopen.bmj.com/site/about/guidelines.xhtml

18. Viveen J, van den Bekerom MPJ, Doornberg JN, et al. Use and outcome of 1,220 primary total elbow arthroplasties from the Australian Orthopaedic Association National Joint Arthroplasty Replacement Registry 2008-2018. *Acta Orthop* 2019;90(6):511-16. doi: 10.1080/17453674.2019.1657342 [published Online First: 20190827]

19. Baghdadi YMK, Veillette CJH, Malone AA, et al. Total elbow arthroplasty in obese patients. *The Journal of bone and joint surgery American volume* 2014;96(9):e70-e70. doi: 10.2106/JBJS.M.00364

20. Riley RD, Moons KG, Hayden JA, et al. Prognostic factor research in healthcare. In: van der Windt DA, Hemingway H, Croft P, et al., eds. Prognosis Research in Healthcare: Concepts, Methods, and Impact: Oxford University Press 2019:0.

21. Damen JAAG, Hooft L. The increasing need for systematic reviews of prognosis studies: strategies to facilitate review production and improve quality of primary research. *Diagnostic and Prognostic Research* 2019;3(1):2. doi: 10.1186/s41512-019-0049-6

22. Moher D, Shamseer L, Clarke M, et al. Preferred reporting items for systematic review and meta-analysis protocols (PRISMA-P) 2015 statement. *Systematic Reviews* 2015;4(1):1. doi: 10.1186/2046-4053-4-1

23. Hemingway H, Croft P, Perel P, et al. Prognosis research strategy (PROGRESS) 1: a framework for researching clinical outcomes. *Bmj* 2013;346:e5595. doi: 10.1136/bmj.e5595 [published Online First: 20130205]

24. Hingorani AD, Windt DAvd, Riley RD, et al. Prognosis research strategy (PROGRESS) 4: Stratified medicine research. *BMJ : British Medical Journal* 2013;346:e5793. doi: 10.1136/bmj.e5793

25. Steyerberg EW, Moons KGM, van der Windt DA, et al. Prognosis Research Strategy (PROGRESS) 3: Prognostic Model Research. *PLOS Medicine* 2013;10(2):e1001381. doi: 10.1371/journal.pmed.1001381

For peer review only

For peer review only - http://bmjopen.bmj.com/site/about/guidelines.xhtml

26. Liebs TR, Splietker F, Hassenpflug J. Is a Revision a Revision? An Analysis of National Arthroplasty Registries' Definitions of Revision. *Clinical orthopaedics and related research* 2015;473(11):3421-30. doi: 10.1007/s11999-015-4255-4

27. Geersing GJ, Bouwmeester W, Zuithoff P, et al. Search filters for finding prognostic and diagnostic prediction studies in Medline to enhance systematic reviews. *PLoS One* 2012;7(2):e32844. doi: 10.1371/journal.pone.0032844 [published Online First: 20120229]

28. Hayden JA, Côté P, Bombardier C. Evaluation of the quality of prognosis studies in systematic reviews. *Ann Intern Med* 2006;144(6):427-37. doi: 10.7326/0003-4819-144-6-200603210-00010

29. Hayden JA, van der Windt DA, Cartwright JL, et al. Assessing bias in studies of prognostic factors. *Ann Intern Med* 2013;158(4):280-6. doi: 10.7326/0003-4819-158-4-201302190-00009

30. IntHout J, Ioannidis JPA, Borm GF. The Hartung-Knapp-Sidik-Jonkman method for random effects meta-analysis is straightforward and considerably outperforms the standard DerSimonian-Laird method. *BMC Medical Research Methodology* 2014;14(1):25. doi: 10.1186/1471-2288-14-25

31. Higgins JPT, Thomas J, Chandler J, et al. Cochrane Handbook for Systematic Reviews of Interventions. 2nd Edition. Chichester (UK): John Wiley & Sons, 2019. 2022.

32. Debray TPA, Moons KGM, Riley RD. Detecting small-study effects and funnel plot asymmetry in meta-analysis of survival data: A comparison of new and existing tests. *Research synthesis methods* 2018;9(1):41-50. doi: 10.1002/jrsm.1266 [published Online First: 2017/11/28]

33. Peters JL, Sutton AJ, Jones DR, et al. Comparison of two methods to detect publication bias in meta-analysis. *Jama* 2006;295(6):676-80. doi: 10.1001/jama.295.6.676

34. Huguet A, Hayden JA, Stinson J, et al. Judging the quality of evidence in reviews of prognostic factor research: adapting the GRADE framework. *Syst Rev* 2013;2:71. doi: 10.1186/2046-4053-2-71 [published Online First: 20130905]

For peer review only - http://bmjopen.bmj.com/site/about/guidelines.xhtml

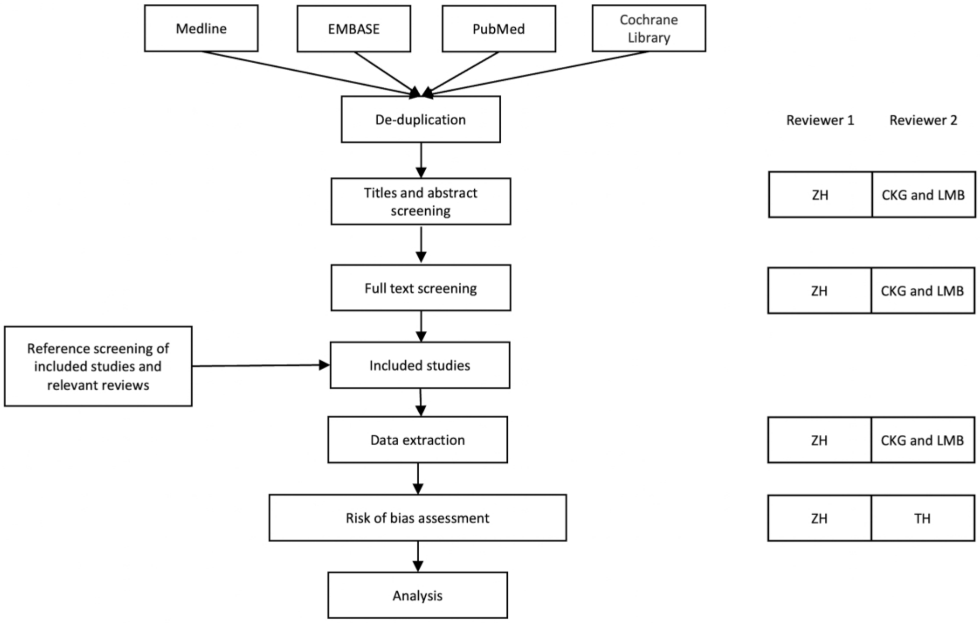

Figure 1: Summary of the systematic review process

394x254mm (500 x 500 DPI)

Supplementary File 1

Electronic search strategies

MEDLINE search - Ovid interface

#	Query
1	exp Arthroplasty, Replacement, Elbow/
2	total elbow replac*.mp. [mp=title, book title, abstract, original title, name of substance word, subject heading word, floating sub-heading word, keyword heading word, organism supplementary concept word, protocol supplementary concept word, rare disease supplementary concept word, unique identifier, synonyms]
3	total elbow arthropl*.mp. [mp=title, book title, abstract, original title, name of substance word, subject heading word, floating sub-heading word, keyword heading word, organism supplementary concept word, protocol supplementary concept word, rare disease supplementary concept word, unique identifier, synonyms]
4	(total and elbow and replac*).ab.
5	(total and elbow and replac*).ti.
6	(total and elbow and arthropl*).ab.
7	(total and elbow and arthropl*).ti.
8	((elbow adj2 arthroplast*) or (elbow adj2 replac*)).mp.
9	1 or 2 or 3 or 4 or 5 or 6 or 7 or 8

EMBASE search- Ovid interface

#	Query
1	exp elbow replacement/ or exp elbow arthroplasty/
2	total elbow replac*.mp. [mp=title, abstract, heading word, drug trade name, original title, device manufacturer, drug manufacturer, device trade name, keyword heading word, floating subheading word, candidate term word]
3	total elbow arthropl*.mp. [mp=title, abstract, heading word, drug trade name, original title, device manufacturer, drug manufacturer, device trade name, keyword heading word, floating subheading word, candidate term word]

For peer review only

4	(total and elbow and replac*).ab.
5	(total and elbow and replac*).ti.
6	(total and elbow and arthropl*).ab.
7	(total and elbow and arthropl*).ti.
8	((elbow adj2 arthroplast*) or (elbow adj2 replac*)).mp.
9	1 or 2 or 3 or 4 or 5 or 6 or 7 or 8
10	limit 9 to conference abstract status
11	9 not 10

PubMed search

ID	Search
#1	"Arthroplasty, Replacement, Elbow"[Mesh]
#2	total elbow replac*<br><br>("total"[All Fields] OR "totaled"[All Fields] OR "totaling"[All Fields] OR "totalled"[All Fields] OR "totalling"[All Fields] OR "totals"[All Fields]) AND ("elbow"[MeSH Terms] OR "elbow"[All Fields] OR "elbow joint"[MeSH Terms] OR ("elbow"[All Fields] AND "joint"[All Fields]) OR "elbow joint"[All Fields] OR "elbow s"[All Fields] OR "elbows"[All Fields]) AND "arthropl*"[All Fields]
#3	total elbow arthropl*<br><br>("total"[All Fields] OR "totaled"[All Fields] OR "totaling"[All Fields] OR "totalled"[All Fields] OR "totalling"[All Fields] OR "totals"[All Fields]) AND ("elbow"[MeSH Terms] OR "elbow"[All Fields] OR "elbow joint"[MeSH Terms] OR ("elbow"[All Fields] AND "joint"[All Fields]) OR "elbow joint"[All Fields] OR "elbow s"[All Fields] OR "elbows"[All Fields]) AND "replac*"[All Fields]
#4	"total"[Title/Abstract] AND "elbow"[Title/Abstract] AND "replac*"[Title/Abstract]
#5	"total"[Title/Abstract] AND "elbow"[Title/Abstract] AND "arthropl*"[Title/Abstract]
#6	(elbow n2 arthropl*) OR (elbow n2 replac*)<br><br>(("elbow"[MeSH Terms] OR "elbow"[All Fields] OR "elbow joint"[MeSH Terms] OR ("elbow"[All Fields] AND "joint"[All Fields]) OR "elbow joint"[All Fields] OR "elbow s"[All Fields] OR "elbows"[All Fields]) AND "n2"[All Fields] AND "arthropl*"[All Fields]) OR (("elbow"[MeSH Terms] OR "elbow"[All Fields] OR "elbow joint"[MeSH Terms] OR ("elbow"[All Fields] AND "joint"[All Fields]) OR "elbow joint"[All Fields] OR "elbow s"[All Fields] OR "elbows"[All Fields]) AND "n2"[All Fields] AND "replac*"[All Fields])
#9	1 or 2 or 3 or 4 or 5 or 6

For peer review only

Cochrane library search

ID	Search
#1	MeSH descriptor: [Arthroplasty, Replacement, Elbow] explode all trees
#2	total elbow replac*
#3	total elbow arthropl*
#4	(Total) AND (Elbow) AND ("replac*"or"arthropl*") (Word variations have been searched)
#5	#1 OR #2 OR #3 OR #4

For peer review only

PRISMA-P (Preferred Reporting Items for Systematic review and Meta-Analysis Protocols) 2015 checklist: recommended items to address in a systematic review protocol*

Section and topic	Item No	Checklist item
**ADMINISTRATIVE INFORMATION**		
Title:		
Identification	1a	Identify the report as a protocol of a systematic review **(Pages 1/Lines 1-3)**
Update	1b	If the protocol is for an update of a previous systematic review, identify as such **(Not an update)**
Registration	2	If registered, provide the name of the registry (such as PROSPERO) and registration number **(Page 14/Line 140 and 336)**
Authors:		
Contact	3a	Provide name, institutional affiliation, e-mail address of all protocol authors; provide physical mailing address of corresponding author **(Page 1/ Lines 6-32)**
Contributions	3b	Describe contributions of protocol authors and identify the guarantor of the review **(Page 14/ Lines 329-335)**
Amendments	4	If the protocol represents an amendment of a previously completed or published protocol, identify as such and list changes; otherwise, state plan for documenting important protocol amendments **(Not an amendment)**
Support:		
Sources	5a	Indicate sources of financial or other support for the review **(Page 14/ Lines 341-346)**
Sponsor	5b	Provide name for the review funder and/or sponsor **(Page 14/ Lines 341-346)**
Role of sponsor or funder	5c	Describe roles of funder(s), sponsor(s), and/or institution(s), if any, in developing the protocol **(Page 14/Page 341-342)**
**INTRODUCTION**		
Rationale	6	Describe the rationale for the review in the context of what is already known **(Pages 5-6/Lines 94-135)**
Objectives	7	Provide an explicit statement of the question(s) the review will address with reference to participants, interventions, comparators, and outcomes (PICO) **(Page 6/Pages 126-135)**
**METHODS**		
Eligibility criteria	8	Specify the study characteristics (such as PICO, study design, setting, time frame) and report characteristics (such as years considered, language, publication status) to be used as criteria for eligibility for the review **(Pages 6-8/ Lines 153-195)**
Information sources	9	Describe all intended information sources (such as electronic databases, contact with study authors, trial registers or other grey literature sources) with planned dates of coverage **(Page 9/ Lines 196-210)**
Search strategy	10	Present draft of search strategy to be used for at least one electronic database, including planned limits, such that it could be repeated **(Supplementary file 1)**
Study records:		
Data management	11a	Describe the mechanism(s) that will be used to manage records and data throughout the review **(Page 10/ Lines 222-223)**

For peer review only

Selection process	11b	State the process that will be used for selecting studies (such as two independent reviewers) through each phase of the review (that is, screening, eligibility and inclusion in meta-analysis). **(Pages 9-10/Lines 212-227)**
Data collection process	11c	Describe planned method of extracting data from reports (such as piloting forms, done independently, in duplicate), any processes for obtaining and confirming data from investigators **(Page 10/Lines 228-240)**
Data items	12	List and define all variables for which data will be sought (such as PICO items, funding sources), any pre-planned data assumptions and simplifications **(Page 10/Lines 231-233)**
Outcomes and prioritization	13	List and define all outcomes for which data will be sought, including prioritization of main and additional outcomes, with rationale **(Page 7/Lines 165-172)**
Risk of bias in individual studies	14	Describe anticipated methods for assessing risk of bias of individual studies, including whether this will be done at the outcome or study level, or both; state how this information will be used in data synthesis **(Pages 10-11/Lines 242-252)**
Data synthesis	15a	Describe criteria under which study data will be quantitatively synthesised **(Page 11/Lines 255-275)**
	15b	If data are appropriate for quantitative synthesis, describe planned summary measures, methods of handling data and methods of combining data from studies, including any planned exploration of consistency (such as I^2, Kendall's τ) **(Pages 11-12/Lines 262-300)**
	15c	Describe any proposed additional analyses (such as sensitivity or subgroup analyses, meta-regression) **(Pages 11/Lines 277-283)**
	15d	If quantitative synthesis is not appropriate, describe the type of summary **planned (Page 12/Lines 291-293)**
Meta-bias(es)	16	Specify any planned assessment of meta-bias(es) (such as publication bias across studies, selective reporting within studies) **(Page 12/Lines 302-308)**
Confidence in cumulative evidence	17	Describe how the strength of the body of evidence will be assessed (such as GRADE) **(Pages12-13/Lines 310-324)**

*** It is strongly recommended that this checklist be read in conjunction with the PRISMA-P Explanation and Elaboration (cite when available) for important clarification on the items. Amendments to a review protocol should be tracked and dated. The copyright for PRISMA-P (including checklist) is held by the PRISMA-P Group and is distributed under a Creative Commons Attribution Licence 4.0.**

From: Shamseer L, Moher D, Clarke M, Ghersi D, Liberati A, Petticrew M, Shekelle P, Stewart L, PRISMA-P Group. Preferred reporting items for systematic review and meta-analysis protocols (PRISMA-P) 2015: elaboration and explanation. BMJ. 2015 Jan 2;349(jan02 1):g7647.

For peer review only
